# Biomimetic Nanocarriers Guide Extracellular ATP Homeostasis to Remodel Energy Metabolism for Activating Innate and Adaptive Immunity System

**DOI:** 10.1002/advs.202105376

**Published:** 2022-04-09

**Authors:** Long Wu, Wei Xie, Yang Li, Qiankun Ni, Peter Timashev, Meng Lyu, Ligang Xia, Yuan Zhang, Lingrong Liu, Yufeng Yuan, Xing‐Jie Liang, Qiqing Zhang

**Affiliations:** ^1^ Institute of Biomedical Engineering & Department of Gastrointestinal Surgery Shenzhen People's Hospital (The Second Clinical Medical College, Jinan University, Jinan University; The First Affiliated Hospital, Southern University of Science and Technology) Shenzhen Guangdong 518020 P. R. China; ^2^ Department of Hepatobiliary & Pancreatic Surgery Zhongnan Hospital of Wuhan University Wuhan Hubei 430071 P. R. China; ^3^ Chinese Academy of Sciences (CAS) Key Laboratory for Biomedical Effects of Nanomaterials and Nanosafety, CAS Center for Excellence in Nanoscience National Center for Nanoscience and Technology of China Beijing 100190 P. R. China; ^4^ University of Chinese Academy of Sciences Beijing 100049 P. R. China; ^5^ Laboratory of Clinical Smart Nanotechnologies, Institute for Regenerative Medicine Sechenov University Moscow 119991 Russia; ^6^ Fujian GTR Biotech Co. Ltd. Fuzhou Fujian 350108 P. R. China; ^7^ Institute of Biomedical Engineering Chinese Academy of Medical Sciences & Peking Union Medical College Tianjin 300192 P. R. China

**Keywords:** AMPK activation, ATP‐adenosine pathway, CD39 inhibition, exosome, immunnometabolic therapy

## Abstract

Metabolic interventions via targeting intratumoral dysregulated metabolism pathways have shown promise in reinvigorating antitumor immunity. However, approved small molecule immunomodulators often suffer from ineffective response rates and severe off‐target toxicity. ATP occupies a crucial role in energy metabolism of components that form the tumor microenvironment (TME) and influences cancer immunosurveillance. Here, a nanocarrier‐assisted immunometabolic therapy strategy that targets the ATP‐adenosine axis for metabolic reprogramming of TME is reported. An ecto‐enzyme (CD39) antagonist POM1 and AMP‐activated protein kinase (AMPK) agonist metformin are both encapsulated into cancer cell‐derived exosomes and used as nanocarriers for tumor targeting delivery. This method increases the level of pro‐inflammatory extracellular ATP (eATP) while preventing the accumulation of immunosuppressive adenosine and alleviating hypoxia. Elevated eATP triggers the activation of P2X7‐NLRP3‐inflammasome to drive macrophage pyroptosis, potentiates the maturation and antigen capacity of dendritic cells (DCs) to enhance the cytotoxic function of T cells and natural killer (NK) cells. As a result, synergistic antitumor immune responses are initiated to suppress tumor progress, inhibit tumor distant metastases, provide long‐term immune memory that offers protection against tumor recurrence and overcome anti‐PD1 resistance. Overall, this study provides an innovative strategy to advance eATP‐driven antitumor immunity in cancer therapy.

## Graphical abstract

CD39 antagonist POM1 and AMPK agonist metformin are both encapsulated into cancer cell‐derived exosomes and used as nanocarriers for tumor targeting delivery. This method increases the level of pro‐inflammatory extracellular ATP while preventing the accumulation of immunosuppressive adenosine and alleviating hypoxia, thereby eliciting strong immune responses to inhibit tumor growth, metastasis, and recurrence.

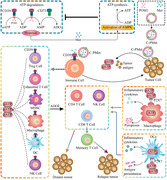

## 1 Introduction

The development of cancer immunotherapy has contributed to a paradigm shift in treating malignancies over the past decade.^[^ ^1^ ^]^ However, therapeutic strategies such as immune checkpoint blockade^[^ ^2^ ^]^ and adoptive T cell transfer^[^ ^3^ ^]^ only benefit a small fraction of patients, with overall clinical response significantly lower in solid tumor therapies.^[^ ^4^ ^]^ Several metabolic and nutrient‐sensing mechanisms orchestrate the behavior of tumor‐infiltrating immune cells to respond to nutrient availability in the tumor microenvironment (TME).^[^ ^5^ ^]^ Notably, the dysregulated metabolic activity of cancer cells can force metabolic stress on immune cells, resulting in local immunosuppression and triggering tumor immune escape.^[^ ^6^ ^]^ Pharmacological interventions targeting metabolic circuits such as aerobic glycolysis,^[^ ^7^ ^]^ amino acids^[^ ^8^ ^]^ and adenosine signaling,^[^ ^9^ ^]^ have shown promise in improving therapeutic efficacy via metabolic reprogramming of the TME, with various clinical trials underway. However, small‐molecular drugs still suffer from severe immune‐related adverse events due to lack of targeting capability,^[^ ^10^ ^]^ while the single immunotherapy approach is unable to produce sufficient therapeutic response.^[^ ^11^ ^]^ In recent years, nanomedicines have provided an alternative way to mitigate these issues owning to their ability to optimize the biodistribution and improve targeted accumulation at desired sites of the systemically administered immunotherapeutic agents.^[^ ^12^ ^]^

Immunometabolic intervention is a unique approach for therapeutic innovation by modulating metabolic pathways such as the ATP‐adenosine axis which plays a crucial role in regulating innate and adaptive immunity.^[^ ^13^ ^]^ Extracellular ATP (eATP) which is released in abundance from stressed or dying cells provides pro‐inflammatory signals to potentiate tumor clearance via promoting macrophage polarization, natural killer (NK) cell activation, and dendritic cells (DCs) targeting and antigen presentation to initiate T cell immunity. These form what we have dubbed the “cancer‐innate immunity cycle”.^[^ ^14^ ^]^ eATP is then degraded into immunosuppressive adenosine through two membrane‐expressed ectonucleotidases: ectonucleoside triphosphate diphosphohydrolase 1 (CD39) and ecto‐5‐nucleotidase (CD73),^[^ ^15^ ^]^ with CD39 driving the hydrolysis of both ATP and ADP to AMP and CD73 efficiently hydrolyzing extracellular AMP, generating adenosine to foster a local immunosuppressive milieu.^[^ ^16^ ^]^ As the rate‐limiting ectoenzyme of eATP degradation, CD39 occupies an apical position in the dynamic balance of accumulation of extracellular ATP and adenosine in the tumor microenvironment.^[^ ^17^ ^]^ And CD39 inhibitors have been demonstrated with the ability to not only abolish adenosine‐driven immunosuppression but also stabilize ATP‐mediated immunostimulation in the TME to boost antitumor responses.^[^ ^18^ ^]^ However, these pharmacologic interventions must be implemented carefully, due to the lack of specificity and toxicity of CD39 antagonisms.^[^ ^19^ ^]^ Furthermore, the hypoxic TME can upregulate the expression of CD39 and CD73, thus promoting ATP degradation and adenosine production through activation of hypoxia‐inducible factor‐1*α* (HIF1*α*), which will inevitably reduce the therapeutic efficacy.^[^ ^20^ ^]^

While attempts to normalize eATP levels at tumor sites could promote antitumor immunity, it might be more beneficial to amply ATP‐dependent cytotoxicity via further improving the ATP concentration in the TME. AMP‐activated protein kinase (AMPK) has achieved widespread acknowledgment as a possible therapeutic target for cancer, which is often correlated with metabolic perturbation.^[^ ^21^ ^]^ Once activated, AMPK rewires cellular metabolism from anabolic process to catabolism through phosphorylating specific key proteins in numerous pathways, thereby minimizing ATP consumption and stimulating ATP generation to re‐establish a more beneficial energy balance.^[^ ^22^ ^]^ Metformin (Met), an antidiabetic drug and a crucial AMPK activator, has shown impressive therapeutic activities against cancer through reprogramming the immune system.^[^ ^23^ ^]^ More importantly, studies have demonstrated that metformin treatment could alleviate intratumoral hypoxia by reducing the oxygen consumption of tumor cells and, inhibiting and destabilizing HIF‐1*α*.^[^ ^24^ ^]^

**Scheme 1:**
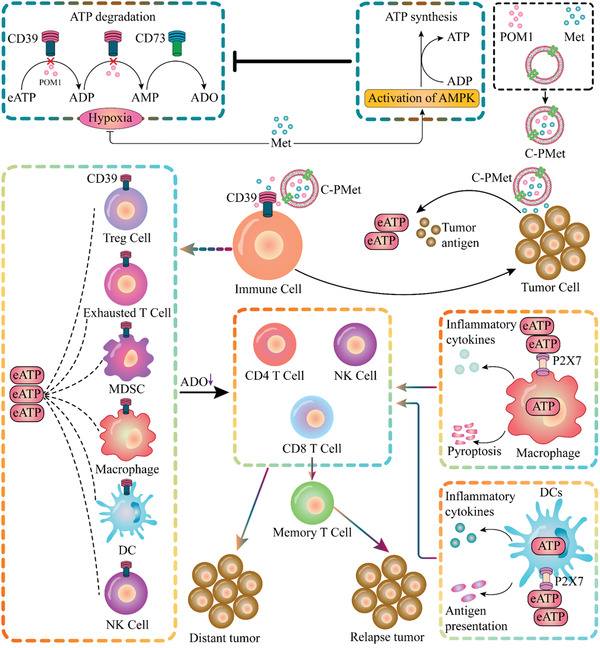
Schematic illustration of anti‐tumor immune responses induced by C‐PMet‐based immunometabolic therapy.

In recent years, exosomes have attracted a lot of attention as nanovesicles for drug delivery.^[^ ^25^ ^]^ Exosomes are extracellular vesicles secreted by cells, which have the advantages of long circulation, good biocompatibility, and low immunogenicity.^[^ ^26^ ^]^ More specifically, Cancer exosomes can express specific proteins and have the ability to target tumors.^[^ ^27^ ^]^ In this work, we drew inspiration from the native properties of nanovesicles and fabricated cancer cell‐derived nanovesicles (denoted as C‐NV) to co‐deliver CD39 inhibitor (sodium polyoxotungstate, POM‐1) and AMPK agonist metformin (denoted as C‐PMet) specifically to tumor sites that enhance immunometabolic therapy, thereby improving antitumor efficiency. The constant release of eATP that is attributed to cell damage or death, coupled with metformin‐mediated AMPK activation to stimulate ATP generation and inhibition of the CD39‐mediated catabolism of eATP by POM‐1 on immune cells, resulted in an increased level of eATP within the TME. High eATP concentrations drive macrophage death by pyroptosis, leading to the secretion of pro‐inflammatory cytokines to enhance the cytotoxic function of effect T and NK cells. Meanwhile, Elevated eATP also potentiates antigen presentation and DCs maturation, further reinvigorating T cell‐mediated immune responses. In addition, by inhibiting CD39 enzymatic activity through POM‐1, the accumulation of adenosine (ADO) also is reduced, thereby attenuating the immunosuppression of NK and T cells. As a result, this C‐PMet‐mediated immunometabolic therapy could exert synergistic antitumor immune responses to suppress tumor progress, prevent distant metastasis, and procure long‐term immune memory protection. (**Scheme** **1**)

## 2 Results and Discussion

### 2.1 Preparation and Characterization of C‐PMet

**Figure 1:**
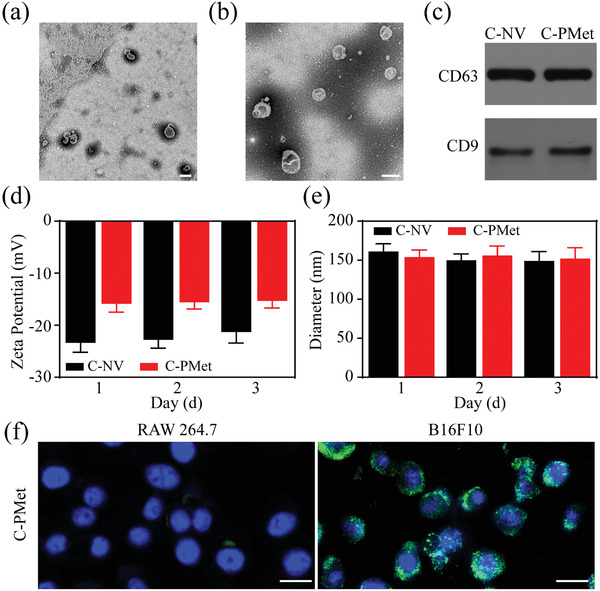
Characterization of C‐PMet. Transmission electron microscopy images of a) C‐NV and b) C‐PMet nanovesicles. Scale bar = 100 nm; c) The expression of CD63 and CD9 from C‐NV and C‐PMet nanovesicles analyzed by western blot analysis; d) Zeta potential values of C‐NV and C‐PMet nanovesicles; e) The hydrodynamic diameter of C‐NV and C‐PMet nanovesicles measured by dynamic light scattering (DLS); f) Representative CLSM images of B16F10 and RAW 264.7 cell after incubation with C‐PMet. Cell nucleus and C‐PMet were labeled with DAPI (blue) and FITC (green), respectively. Scale bar = 20 µm.

C‐NV was acquired from the supernatants of B16F10 murine melanoma cells. Transmission electron microscopy (TEM) revealed that isolated C‐NV ≈150 nm and had a saucer‐like shape (**Figure** **1**a). To obtain precise delivery of drugs to the tumor tissue, we used electroporation to send the water‐soluble drugs (POM1 and Met) inside the C‐NV. As shown in Figure 1b, the presence or absence of drug loading had little effect on the morphology of C‐NV. Western blot analysis further demonstrated that these C‐PMet nanovesicles were phenotypically similar to C‐NV, as they were shown expressing typical exosomal biomarkers, including CD63 and CD9 (Figure 1c). To determine whether the drugs were sent into the C‐NV, we analyzed the zeta potential of C‐NV and C‐PMet and found that the zeta potential of C‐PMet was obviously higher compared with C‐NV, which demonstrated that the POM1 and Met had been successfully loaded into the C‐NV (Figure 1d). Met electroporation efficiency values were calculated under different initial Met concentrations (Figure S1a, Supporting Information), revealing that packaging efficiency slowly rose as starting Met concentrations increased, until reaching a maximum value of 79.2%. Additionally, C‐PMet showed sustained drug release in various pH conditions (Figure S1b, Supporting Information). In addition, no significant changes were observed either in the size or zeta potential of C‐PMet and C‐NV nanovesicles following 3 days of storage in PBS at 4 ℃, indicating that these particles were highly stable (Figure 1d,e). Since C‐NV was derived from B16F10 cancer cells, we wondered whether C‐PMet was being internalized by achieving cancer cell targeting. To investigate the tumor cell‐targeting capacity of C‐PMet nanovesicles in vitro, we used RAW 264.7 cells as a control group and showed that C‐PMet was abundantly enriched around B16F10 cells (Figure 1f). The antitumor activity of C‐PMet on B16F10 cells was further evaluated. As shown in Figure S2 (Supporting Information), C‐PMet demonstrated moderate cytotoxicity on B16F10 cells in vitro. These results suggested that C‐PMet was an ideal platform for targeting tumor cells.

### 2.2 In Vitro Immune Activation

**Figure 2:**
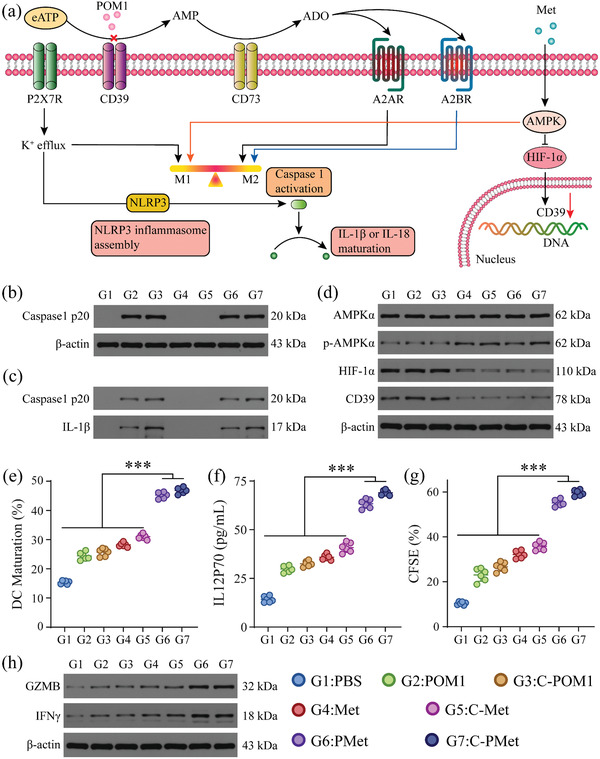
In vitro C‐PMet‐mediated immune activation. a) Schematic illustration of immune regulation induced by POM1 and Metformin; b, c) LPS‐primed bone marrow‐derived macrophages (BMDMs) were incubated with PBS, POM1(free POM1), C‐POM1(C‐NV loaded with POM1), Met (free metformin), C‐Met (C‐NV loaded with metformin), PMet (free POM1 and metformin) and C‐PMet (C‐NV loaded with POM1 and metformin). Western blot analysis of caspase1 p20 from cell lysates (b), and caspase1 p20 and IL‐1*β* from culture supernatants (c); d) Western blot analysis of AMPK*α*, p‐AMPK*α*, HIF1*α*, and CD39 from BMDMs; e) In vitro BMDCs maturation rate (gated on CD11c^+^CD80^+^CD86^+^); f) Production of IL‐12p70 secreted by treated BMDCs; g) The proliferation of CFSE‐labeled CD8^+^ T lymphocytes; h) Western blot analysis of GZMB and IFN‐*γ* from T cells. Data are presented as mean ± s.d. (n = 6). ***p < 0.001. One‐way ANOVA followed by Tukey post‐hoc test.

Within the tumor microenvironment, high levels of eATP can trigger inflammation responses via the activation of P2 purinergic receptors (mainly P2X7R), which can be hydrolyzed to adenosine (ADO) by the cascaded action of CD39 and CD73 to induce an immunosuppressive microenvironment through binding to A2A or A2B receptors (**Figure** **2**a). Studies have demonstrated that ATP accumulation resulting from CD39 inhibition favors macrophages polarizing into a pro‐inflammatory M1 phenotype,^[^ ^28^ ^]^ and metformin also has been shown to have the ability to repolarize anti‐inflammatory M2‐like macrophage to M1 phenotype through activating the AMPK pathway.^[^ ^29^ ^]^ We wondered whether POM1 and metformin could work synergistically to further promote macrophage polarization. We examined the interactions of our C‐PMet nanovesicles with mouse bone marrow‐derived macrophages (BMDMs). As shown by the flow cytometry experiments, the proportion of M1 macrophages was increased after being treated with C‐POM1 or C‐Met, while the combined treatment could further contribute to polarization (Figures S4 and S5, Supporting Information). Meanwhile, macrophage markers were also detected by RT‐qPCR. After C‐PMet treatment, the mRNA levels of iNOS, IL‐6, and TNF*α* (markers for M1 macrophage) were obviously improved (Figure S6, Supporting Information), whereas the mRNA levels of Arg1, IL‐10, and TGF*β* (markers for M2 macrophage) were dramatically decreased (Figure S7, Supporting Information). Mechanistically, CD39 inhibition‐liberated eATP accumulation induced NLRP3 inflammasome and caspase1 activation, leading to the maturation and release of pro‐inflammatory cytokine IL1*β*, demonstrated by western blot analysis (Figure 2b,c). Met was also proven to alleviate hypoxia via activating AMPK to inhibit hypoxia‐inducible factor‐1*α* (HIF‐1*α*).^[^ ^24^, ^30^ ^]^ The expression levels of AMPK*α* and HIF‐1*α* in treated BMDMs were measured and as we expected, C‐Met and C‐PMet treatment boosted AMPK*α* phosphorylation while decreasing HIF‐1*α* expression. No obvious difference was observed in C‐POM1 treated group. C‐Met and C‐PMet treatment also reduced the expression of CD39 in BMDMs, associated with the inhibition of HIF‐1*α* as hypoxia plays a crucial role in the induction of CD39 expression (Figure 2d).

DCs could uptake and present antigens to T lymphocytes, eliciting a strong antigen‐specific adaptive immune response.^[^ ^31^ ^]^ A transwell co‐culture system was established to elucidate immune stimulation abilities of C‐PMet toward DCs separated from the bone marrow of C57BL/6 mouse in vitro. Starting levels of co‐stimulation molecules, CD80 and CD86 (markers for DCs maturation), were analyzed by flow cytometry. As demonstrated, B16F10 cells treated by C‐PMet and PMet advanced the DCs’ maturation to a higher level than other groups (Figure 2e and Figure S8, Supporting Information). Consistently, C‐PMet and PMet induced the highest productions of TNF‐*α* (Figure S9, Supporting Information) and IL‐12p70 (Figure 2f) by treated BMDCs. Given the main force of T cells for adaptive immune response,^[^ ^32^ ^]^ the treated BMDCs were further co‐incubated with mouse spleen‐derived CD8^+^ T cells. C‐PMet and PMet pre‐treated BMDCs could significantly increase the proliferation of CD8^+^ T cells (Figure 2g) while enhancing the function of CD8^+^ lymphocytes. This was demonstrated by the improved expression of Granzyme B (GZMB) and IFN‐*γ* analyzed by western blot analysis (Figure 2h and Figure S3, Supporting Information). All results demonstrated C‐PMet nanovesicles could potentiate DCs maturation, further reinvigorating T cell activation.

### 2.3 In Vivo Immunometabolic Therapy

**Figure 3:**
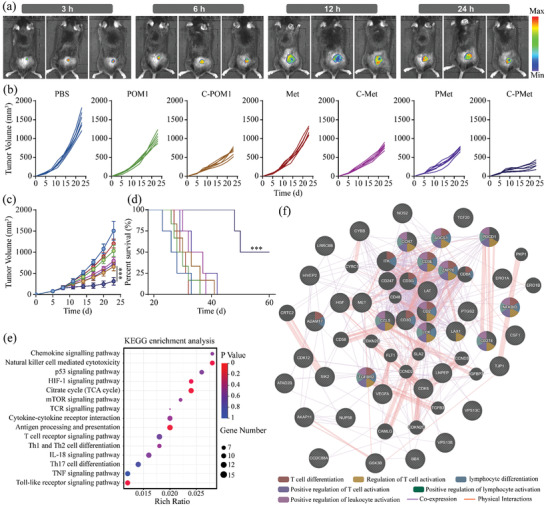
In vivo anti‐tumor activity of C‐PMet‐mediated immunometabolic therapy. a) Fluorescence imaging of mice with B16F10 tumors at different time points following the intravenous injection of C‐PMet@Cy5.5; Growth curves of b) Individual and c) average B16F10 tumors; d) Survival curves of mice in different groups. Data are presented as mean ± s.d. (n = 6). ***p< 0.001. One‐way ANOVA followed by Tukey post‐hoc test; e) KEGG analysis of immune response‐associated pathways elicted by C‐PMet; f) Gene‐MANIA for forecasting interactions among differential expressed genes.

As C‐PMet‐based treatment demonstrated efficient functions to induce robust immunological responses in vitro, we wondered whether such a combinational treatment could enhance the therapeutic efficacy in vivo. We first investigated the tumor‐targeting ability of C‐PMet in B16F10 tumor‐bearing mice. A remarkable accumulation at the tumor sites was noticed after 12 h following the C‐PMet injection (**Figure** **3**a). Then we evaluated the efficacy of C‐PMet‐mediated immunometabolic therapy. As demonstrated in Figure 3b, c, while both C‐POM1 and C‐Met significantly delayed the growth of tumors, the combined treatment (C‐PMet) inhibited the tumor growth even further. C‐PMet also prolonged the survival of treated mice in the 60‐day observed period (Figure 3d). We also measured the body weight fluctuation in the mice and as predicted, no obvious difference was observed among all treated mice (Figure S10, Supporting Information). To further investigate the toxicity of C‐PMet in vivo, blood and major organs were obtained from the treated mice for blood biochemistry and H&E staining assessments. The liver and kidney function index revealed that C‐PMet barely caused any injury (Figure S11, Supporting Information), no apparent histopathological abnormalities were shown in major organs (Figure S12, Supporting Information), indicating that this therapy strategy had good biocompatibility for treated mice.

To understand the mechanism of C‐PMet‐mediated antitumor immune responses, we evaluated the transcriptomic feature of the B16F10 tumor tissues after treatment. The Kyoto Encyclopedia of Genes and Genomes (KEGG) analysis was first performed to identify immune‐associated activation pathways after C‐PMet therapy compared with PBS. From the KEGG enrichment analysis, we found that the Cytokine‐cytokine receptor interaction and T cell receptor signaling pathways showed the most significant differences (Figure 3e). Besides, Gene‐MANIA was performed to predict gene interactions. As it demonstrated, most of these genes were related with co‐expression and shared protein domains (Figure 3f). A large number of genes related with cytokine activity (26 of 136) were activated. What's more, changes of a gene cluster associated with T‐cell activation (15 of 108), proliferation (18 of 124), and leukocyte (18 of 228), were also observed. All data illustrated the impressive immune activation capacity of C‐PMet.

### 2.4 The Mechanism of C‐PMet Mediated Antitumor Responses

**Figure 4:**
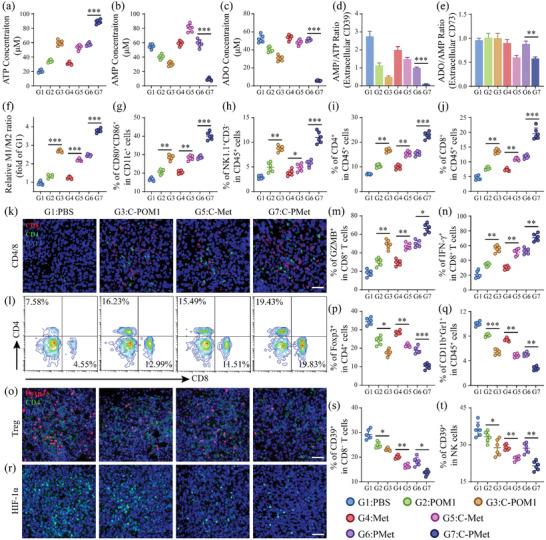
C‐PMet strategy harnesses innate immune system to initiate and activate adaptive T‐cell immunity. a–c) ATP (a), AMP (b) and ADO (c) concentrations in tumors after different treatments; Enzymatic activities of d) CD39 and e) CD73 measured by HPLC analysis; f) Ratio of M1‐like (CD45^+^CD11b^+^F4/80^+^CD80^+^) / M2‐like (CD45^+^CD11b^+^F4/80^+^CD206^+^) macrophages in the tumor tissues of B16F10‐tumor‐bearing mice after indicated treatments; g) The frequency of mature DCs in tumor‐draining lymph nodes of C57BL/6 mice (gated on CD45^+^CD11c^+^CD80^+^CD86^+^); h) Quantitative analysis of NK cells (CD45^+^NK1.1^+^CD3^–^) in tumor tissues; i) Proportions of tumor‐infiltrating CD4^+^ T cells in different groups (gated on CD45^+^); j) Percentages of CD8^+^ T cells in different groups (gated on CD45^+^); k) Immunofluorescence images of residual tumors demonstrating CD4^+^ T (green) and CD8^+^ (red) T cells infiltration. Scale bars = 50 µm; l) Flow cytometry plots of CD4^+^ and CD8^+^ T cells in tumors (gated on CD45^+^); m, n) Proportions of GZMB (m) and IFN‐*γ*‐positive (n) CD8^+^ T cells within tumors; o) Immunofluorescence images of residual tumors showing CD4^+^ (green) Foxp3^+^ (red) Treg cells infiltration. Scale bars = 50 µm; p) Proportions of tumor‐infiltrating Tregs after various treatments (gated on CD4^+^); q) Quantification of MDSC (CD45^+^CD11b^+^Gr‐1^+^) in tumor tissues; r) Immunofluorescence images of residual tumors showing HIF‐1*α*. Scale bars = 50 µm; s, t) Quantification of CD8^+^CD39^+^ T (s) and CD39^+^ NK (t) cells in B16F10 tumors. Data are presented as mean ± s.d. (n = 6). *p < 0.05, **p < 0.01, ***p < 0.001. One‐way ANOVA followed by Tukey post‐hoc test.

Firstly, we validated the effects of C‐PMet on levels of ATP, AMP, and ADO in the tumor tissues. As shown in **Figure** **4**a, the average ATP concentration in the PBS group was 20.3 µM, and treatment with C‐PMet significantly elevated the ATP concentration to 89.2 µM. Conversely, C‐PMet reduced ADO concentrations from 54.6 to 5.6 µM (Figure 4c). C‐PMet treatment also inhibited the ectoenzymatic activity of CD39 (convert ATP to AMP) and CD73 (convert AMP to ADO) (Figure 4d,e). Innate immune responses are essential to initiate and maintain adaptive immunity by participating in the processes of T‐cell priming, expansion, and infiltration at the tumor site, called cancer–innate immunity cycle.^[^ ^33^ ^]^ To investigate the effects of high concentration eATP on tumor immune microenvironment, components of innate immunity including myeloid such as macrophages and DCs, and NK cells were analyzed by collecting the tumor tissues and inguinal lymph nodes from nanovesicle‐injected B16F10‐tumor‐bearing mice. Tumor‐associated macrophages (TAMs) are predominant populations of myeloid cells within the tumor immune microenvironment and grow to be M2‐like phenotypes, prohibiting the infiltration of tumor‐specific cytotoxic T cells and causing resistance to anti‐PD1 therapy.^[^ ^34^ ^]^ As demonstrated in Figure 4f, there was an increased ratio of M1 and M2‐like TAMs in the tumors treated with C‐PMet, by 3.95‐fold relative to that of PBS‐injected mice. Meanwhile, the number of B16F10 tumor‐infiltrating macrophages was obviously reduced (Figure S13, Supporting Information) and the level of intratumor IL‐18 was significantly increased (Figure S14, Supporting Information) under C‐PMet treatment conditions, supporting that C‐PMet‐liberated eATP induced macrophage pyroptosis and triggered inflammasome activation with inflammatory cytokine release. Tumor‐draining lymph nodes were harvested for analyzing expression levels of DCs markers (CD11c, CD80, CD86) by flow cytometry analysis. Interestingly, C‐PMet‐based therapy showed the highest level of DCs’ maturation among all groups, which was 2.6‐fold higher than the PBS‐injected group (Figure 4g). In addition, the population of NK cells (NK1.1^+^CD3^–^) in the tumor tissues of C‐PMet‐injected mice was also higher than control treatment groups (Figure 4h). These data demonstrated that C‐PMet‐mediated therapy could significantly induce the activation of innate immune cells.

To examine the effectiveness of the C‐PMet‐based immunometabolic therapy used to stimulate proliferation and functional activation of intratumoral CD8^+^ cells, we harvested and studied tumor‐infiltrating T lymphocytes from tumor tissues. As revealed by immunofluorescence staining and compared with control groups, the residual tumors from C‐PMet treated mice were greatly infiltrated with CD4^+^ and CD8^+^ T cells (Figure 4k). The percentages of CD4^+^ and CD8^+^ lymphocytes after C‐PMet‐based therapy increased rapidly to 24.6 and 22.9 percent, appearing to be higher than in groups treated with C‐POM1 or C‐Met treatment alone, even the group treated with PMet (Figure 4i,j,l). Furthermore, compared with control treatments, proliferation of CD8^+^CTL was higher within the tumors of C‐PMet treatment by measuring the percentage of Ki67 (Figure S15, Supporting Information). Meanwhile, the C‐PMet therapy also increased the proportions of both GZMB and IFN‐*γ*‐positive cytotoxic CD8^+^ T cells (Figure 4m, n), which explains the improved tumor‐killing ability of intratumoral CD8^+^ T cells. Since the immunosuppressive cells in the TME plays an important role in manipulating T cell responses against tumors, the infiltration of regulate T cells (Tregs) and myeloid‐derived suppressor cells (MDSCs) in the tumors were also measured. Tumor‐infiltrating CD4^+^ Foxp3^+^ T cells were analyzed after co‐staining with CD4 and Foxp3. We discovered that the percentage of Treg cells greatly decreased in the C‐PMet group (Figure 4o, p). Similarly, the frequency of MDSCs was remarkably lower after treating the mouse with C‐PMet (Figure 4q). As immunofluorescence staining revealed, the expression of HIF‐1*α* was significantly scavenged in both C‐Met and C‐PMet groups (Figure 4r). More importantly, a much‐reduced frequency of exhausted T cells (CD8^+^CD39^+^), markers for T cell dysfunction, was observed in CD8^+^ clusters after C‐PMet therapy (Figure 4s). At the same time, the percentage of CD39^+^ NKs was also lower, indicating that while there are more NKs in the C‐PMet treatment group, the cells are more stable and functional (Figure 4t).

All the above observations, when analyzed together, indicated that the C‐PMet‐based immunometabolic therapy produced significant antitumor effects by triggering a strong T cell‐mediated immunological response and modulating the tumor immune‐suppressing microenvironment.

### 2.5 C‐PMet Combined with Anti‐PD1 Treatment to Inhibit Tumor Metastasis

**Figure 5:**
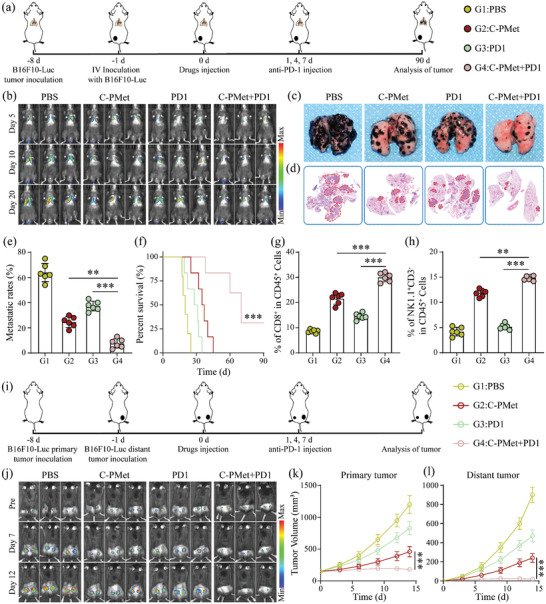
The anti‐tumor metastasis activity of C‐PMet combined with anti‐PD1 treatment. a) Schematic illustration of therapy schedule to inhibit tumor lung metastasis; b) Bioluminescence imaging of treated mice at indicated time points; c) Representative lung photographs; d) H&E staining of lung tissue slices. Scale bars = 50 µm; e) Metastatic rates of pulmonary nodules after various treatments; f) Survival curves in different groups; g,h) Quantitative analysis of CD8^+^ T and NK cells in lung tissues (gated on CD45^+^ cells); i) Schematic illustration of treatment schedule to inhibit tumor distant metastasis; j) Bioluminescence imaging of mice at indicated time points; k) Primary and l) distant tumor growth curves after indicated treatments. Data are presented as mean ± s.d. (*n* = 6). **p < 0.01, ***p < 0.001. One‐way ANOVA followed by Tukey post hoc test.

Inspired by the results that C‐PMet could covert the aggressive B16F10 melanoma model from “cold” tumor into “hot” one (that was more likely to respond to anti‐PD1 therapy) by enhancing the infiltration of CD8^+^ lymphocytes and reshaping the immunosuppressive microenvironment, we next investigated the combined therapeutic efficacy of C‐PMet and PD‐1 blockade in a more aggressive tumor model: B16F10‐Luc cells were intravenously injected into C57BL/6 mice through their tail veins 7 days later after the primary tumors were established (**Figure** **5**a). C‐PMet combined with anti‐PD‐1 treatment achieved systemic therapeutic efficacy to inhibit tumor lung‐metastasis as demonstrated by bioluminescence imaging (Figure 5b). At the endpoints, the lungs were harvested to determine the therapeutic efficacy of indicated treatment strategies. Photographs and H&E staining indicated that: compared with groups of PBS, C‐PMet and PD1 where obvious metastatic tumors nodules were detected in the lungs, fewer tumor lesions were shown in the group treated with C‐PMet and anti‐PD‐1 therapy (Figure 5c–e). Meanwhile, survival of the mice (n = 6/group) in all groups following indicated treatments was closely monitored. Compared with PBS and anti‐PD1 groups, C‐PMet therapy promoted the survival time of treated mice, and the combination of C‐PMet and anti‐PD1 further significantly prolonged the survival rate of treated mice in the 90‐day observation period (Figure 5f). During tumor metastasis, NK cells and cytotoxic T lymphocytes (CTLs) are two main types of effector cells to trigger innate and adaptive immune responses that target and eradicate CTCs directly, while other immune cells usually affect metastatic cascade through modulating the function of the formers.^[^ ^35^ ^]^ As indicated, the frequency of NK cells was remarkable when evaluated in the C‐PMet+PD1 group (Figure 5h), consistently enhancing infiltration of CD8^+^ T in lung tissues compared with other groups (Figure 5g).

We further established a dual‐tumor model to determine the anti‐metastatic ability by subcutaneously injecting B16F10‐Luc cells into the right (as primary tumors) and left (distant tumors) frank of mice (Figure 5i). The primary and distant tumors were both significantly restrained in the C‐PMet+PD1 group (Figure 5j–l), meanwhile treatment with C‐PMet+PD1 also obviously enhanced the population of NK and CD8^+^ T cells in the distant tumors (Figures S16 and 17, Supporting Information). In conclusion, these results demonstrated that C‐PMet‐assisted immunometabolic therapy in combination with anti‐PD1 treatment could synergistically suppress tumor metastasis.

### 2.6 Long‐Term Immune Memory Protection Against Tumor Recurrence

**Figure 6:**
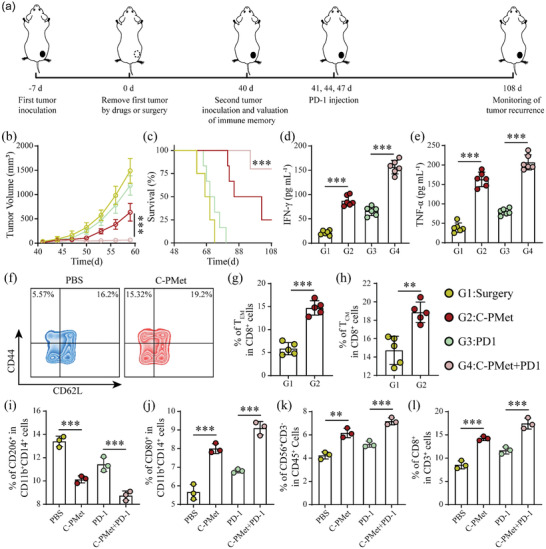
Durable immunological effect against rechallenged tumors elicited by C‐PMet and improved TME induced by the combination of C‐PMet with anti‐PD1 antibody in organotypic tumor slices from colon cancer tissues. a) Schematic illustration of the combination of C‐PMet and anti‐PD1treatment to prohibit tumor recurrence; b) Average tumor volume curves of recurrence tumors; c) Survival curves of mice after rechallenged with B16F10 tumors; d,e) Cytokine levels of IFN‐*γ* and TNF‐*α* in sera from mice on day 47. f) Representative flow cytometry plots and g, h) quantification of effector memory T cells (T_EM_) (CD3^+^CD8^+^CD44^+^CD62L^–^) and central memory T cells (T_CM_) (CD3^+^CD8^+^CD44^+^CD62L^+^) in the spleen on day 40 before re‐inoculating mice with secondary tumors. Data are presented as mean ± s.d. (n = 6). **p < 0.01, ***p < 0.001. i–l) Percentages of M2‐like macrophages (i), M1‐like macrophages (j), NK cells (k), and CD8^+^T cells (l) in the organotypic slices after treatment with PBS, C‐PMet, anti‐human PD‐1 antibody, or C‐PMet and anti‐human PD‐1 antibody in the presence of PBMCs for 36h, respectively. Data are presented as mean ± s.d. (n = 3). **p < 0.01, ***p < 0.001. Comparison between two groups was performed using unpaired two tailed Student's *t*‐test. For comparison of multiple groups, One‐way ANOVA followed by Tukey post hoc test.

To evaluate the durable immune memory induced by C‐PMet against tumor relapse, after the primary tumors were completely eliminated by surgery resection or C‐PMet, the mice were rechallenged with B16F10 cells on day 40 (**Figure** **6**a). The tumor growth in mice with primary tumors removed by C‐PMet therapy was obviously delayed. More importantly, most mice in group 4 displayed complete tumor regression and survived with an extra three doses of anti‐PD1 treatment as shown in Figure 6b, c.

To further evaluate the immunological memory, the spleens from mice treated with C‐PMet or surgery were harvested and examined by flow cytometric analysis on day 40. As it demonstrated, C‐PMet treatment significantly increased the populations of memory T cells including effector (T_EM_) and central (T_CM_) cells in group2 (Figure 6f–h). We also measured the cytokines in sera from treated mice on day 47. Consistent with the increasing population of memory T lymphocytes, the levels of IFN‐*γ* and TNF‐*α* were increased in groups 2 and 4(Figure 6d,e). These results verified the efficiency of the immune memory elicited by C‐PMet‐mediated immunometabolic therapy.

### 2.7 C‐PMet Triggers Robust Antitumor on Patient‐Derived Tumor Slice

To investigate whether C‐PMet based immunometabolic therapy combined with anti‐PD‐1 treatment could enhance antitumor activity in preclinical models, we established an ex‐vivo organotypic tumor slice culture derived from fresh human colon cancer tissues (Figure S18, Supporting Information). As it demonstrated, while C‐PMet markedly induced apoptosis of tumor cells (Figure S19, Supporting Information), this treatment also reshaped the macrophage polarization by decreasing the population of M2‐like TAMs (Figure 6i) and increasing M1 phenotype (Figure 6j), and promoted the infiltration of NK (Figure 6k) and CD8^+^ T cells (Figure 6l), compared with PBS or even anti‐PD‐1antibody alone. However, the combination of C‐PMet and PD‐1 blockade further promoted apoptosis induction, enhanced the repolarization of M2‐like macrophages to M1 phenotype, and increased the recruitment of effector NK and CD8^+^ T cells in tumor tissues (Figure 6i–l). These findings further suggested that C‐PMet could indeed remodel the TME and enhance the antitumor efficacy of anti‐PD‐1 antibody therapy.

## 3 Conclusion

We developed a cancer exosome nanovesicles‐assisted therapeutic strategy to specifically deliver two biocompatible components for local immunometabolic therapy: a CD39 inhibitor POM1 and AMPK agonist metformin. Both components exhibited functions to elicit strong tumor‐specific immune responses for synergistically inhibiting tumor growth, achieve significant abscopal effects to inhibit tumor distant metastases, induce long‐term immune memory to protect mice against recurrence, and sensitize tumors to anti‐PD1 therapy. The mechanism can be concluded as follows: Targeting the ATP‐adenosine pathway via pharmacological blockade of CD39 and activation of AMPK not only promotes the accumulation of pro‐inflammatory eATP but also decreases the levels of immunosuppressive adenosine, thereby triggering an ATP‐dependent antitumor immunity by inducing pyroptosis in macrophages and improving DCs’ maturation, which allows for the activation of tumor‐specific CD8^+^ T and NK cells. This strategy shows potential for clinical alternatives in patients with a limited response rate or for maintaining the longevity of immune response in those who have benefited from clinical benefit treatments with conventional immunotherapy alone.

To discover novel immunotherapeutic strategies focused on local treatment, the connection between tumor‐specific immune responses and biomaterials requires deeper understanding. This can be achieved through carefully controlling immune responses by modifying biomaterials or modulating the tumor immunosuppressive microenvironment.

## 4 Experimental Section

Detailed materials and methods can be found in the Supporting Information.

## Conflict of Interest

The authors declare no conflict of interest.

## Supporting information

Supporting Information

## Data Availability

Research data are not shared.
