# Supplementary material for: Biomimetic Nanocarriers Guide Extracellular ATP Homeostasis to Remodel Energy Metabolism for Activating Innate and Adaptive Immunity System
Source: Adv Sci (Weinh). 2022 Apr 9;9(17):2105376. doi: 10.1002/advs.202105376 (PMC9189650; doi:10.1002/advs.202105376)
Supplement: Supplementary file 1 — Supporting Information [file ADVS-9-2105376-s001.pdf]

ADVANCED SCIENCE
Open Access

Supporting Information

for *Adv. Sci.*, DOI 10.1002/advs.202105376

Biomimetic Nanocarriers Guide Extracellular ATP Homeostasis to Remodel Energy Metabolism for Activating Innate and Adaptive Immunity System

Long Wu, Wei Xie, Yang Li, Qiankun Ni, Peter Timashev, Meng Lyu, Ligang Xia, Yuan Zhang, Lingrong Liu, Yufeng Yuan, Xing-Jie Liang* and Qiqing Zhang**

Supporting Information

Biomimetic Nanocarriers Guide Extracellular ATP Homeostasis to Remodel Energy Metabolism for Activating Innate and Adaptive Immunity System

Long Wu,[1, 2, #] Wei Xie,[2, #] Yang Li,[1, #] Qiankun Ni,[3, 4] Peter Timashev,[5] Meng Lyu,[1] Ligang Xia,[1] Yuan Zhang,[6] Lingrong Liu,[7] Yufeng Yuan,[2, *] Xing-Jie Liang,[3, 4, *] and Qiqing Zhang[1, 7, *]

[1.] Institute of Biomedical Engineering & Department of Gastrointestinal Surgery, Shenzhen People's Hospital (The Second Clinical Medical College, Jinan University; The First Affiliated Hospital, Southern University of Science and Technology), Shenzhen, Guangdong, 518020, P. R. China.

[2.] Department of Hepatobiliary & Pancreatic Surgery, Zhongnan Hospital of Wuhan University, Wuhan, Hubei, 430071, P. R. China.

[3.] Chinese Academy of Sciences (CAS) Key Laboratory for Biomedical Effects of Nanomaterials and Nanosafety, CAS Center for Excellence in Nanoscience, National Center for Nanoscience and Technology of China, Beijing, 100190, P. R. China.

[4.] University of Chinese Academy of Sciences, Beijing, 100049, P. R. China.

[5.] Laboratory of Clinical Smart Nanotechnologies, Institute for Regenerative Medicine, Sechenov University, Moscow, 119991, Russia.

[6.] Fujian GTR Biotech Co. Ltd., Fuzhou, Fujian, 350108, P. R. China.

[7.] Institute of Biomedical Engineering, Chinese Academy of Medical Sciences & Peking Union Medical College, Tianjin, 300192, P. R. China

[#] These three authors contributed equally to this work.

* Authors to whom correspondence should be addressed.

Emails: zhangqiq@126.com; liangxj@nanoctr.cn; yuanyf1971@whu.edu.cn

Materials and Methods

Materials

All chemicals were purchased from Sigma-Aldrich unless otherwise specified. PD-1mAbs were purchased from Bio-X-cell (Clone, RMP1-14). POM-1was purchased from MCE.

Cell culture

The murine melanoma cell lines B16F10 and macrophages RAW264.7, and the human colon cancer cells HCT116 were purchased from the Cell Bank of Shanghai, Chinese Academy of Sciences. B16F10-Luc cell lines were established by transfection of B16F10 with vectors carrying luciferase and puromycin resistance gene. B16F10, B16F10-Luc and RAW264.7 cells were cultured in DMEM medium supplemented with 10% FBS and 1% antibiotics (penicillin/streptomycin). HCT 116 cells were maintained in McCoy's 5A medium containing 10% FBS and 1% antibiotics (penicillin/streptomycin). Bone marrow-derived macrophages (BMDMs) and bone marrow-derived dendritic cells (BMDCs) were prepared following the steps blow. C57BL/6 mice were sacrificed and bone marrow cells were isolated from leg bones. To generate BMDMs, the isolated bone marrow cells were resuspended and maintained in complete RPMI medium supplemented with 10% FBS, 1% antibiotics (penicillin/streptomycin) and M-CSF (20 ng/mL) for 7 days. To generate BMDCs, the isolated bone marrow cells were resuspended and cultured in complete RPMI medium containing 10% FBS, 1% antibiotics (penicillin/streptomycin) and GM-CSF (20 ng/mL) for 7 days. All the cells were maintained in a normal oxygen incubator at 37 °C

with 5% CO_2. Hypoxia condition was performed in a hypoxic cell incubator with 2% O_2, 5% CO_2 and balanced nitrogen.

Animals

Male C57BL/6 mice (6 to 7 weeks of age) were obtained from the Chinese Academy of Medical Science (Beijing, China). All animal handling and experimental procedures were approved by the Center for Animal Experiments/Animal Biosafety Level 3 Laboratory of Wuhan University.

Preparation and characterization of exosome/C-PMet

The exosomes harvested from B16F10 tumor cells were prepared according to the manufacturer's protocol of Exosome Isolation Reagent (RIBOBIO biotechnology co. LTD, China). To load POM1 and Met into the exosomes, the exosomes (0.5 mg) and drugs (50 μg in 10 μL DMSO) were mixed in 250 μL PBS in 0.4 cm cuvette (Bio-Rad). Electroporation was then carried out at 250 V and 350 μF on a Bio-Rad Gene Pulser X-cell Electroporation System. After electroporation, the mixture was incubated at 37 °C for 30 min to allow the recovery of the membrane of the electroporated exosomes. The obtained solution was centrifuged at 8000 g for 5min to remove the precipitate (redundant POM1 and Met) and the C-PMet in supernatant were collected by using Exosome Isolation Reagent again and stored at 4 °C for further use. The size and size distribution of C-PMet were measured by dynamic light scattering. The morphology structures of C-NV and C-PMet were observed by the TEM (JEOL-2100). The amount of Met loaded into C-NV and release from C-PMet curves followed by quantification *via* an HPLC system.

Western blot analysis

C-NV and C-PMet were prepared in SDS sample buffer and loaded into 10-12% SDS polyacrylamide gel. The proteins electrophoresed were then transferred onto polyacrylamide fluoride (PVDF) membranes. The membranes were further blocked with 5% skim milk for 1 h and incubated with primary antibodies against CD9 (ab223052, Abcam) and CD63 (ab217345, Abcam) at 4 °C over night. The PVDF membranes were then incubated with second antibody and detected using a Clarity^{TM} Western ECL Substrate (Bio-Rad).

In vitro cancer targeting study

B16F10 and RAW 264.7 cells were seeded in 24-well plates and cultured for 12 h. Then 10 μL of C-PMet (FITC) were added into the culture medium. The cells were incubated at 37 °C, 5% CO_2 for 2 h, and washed with PBS for three times. Afterwards, the cells were further fixed with PFA for 30 min at room temperature, and then stained with DAPI and imaged by using a confocal laser scanning microscope (CLSM; IX81, Olympus, Japan).

In vitro toxicity of C-PMet

The toxicity was measured by MTT assay. B16F10 cells were seeded in 96-well plates at a density of 5×10^3 cells per well and incubated for 24 h. Afterwards, B16F10 cells were incubated for 4 different groups: (1) C-NV (2) C-Met (3) C-POM1 (4) C-PMet. At the end of the incubation, 5 mg/mL MTT PBS solution was added, and the plate was incubated for another 4 h. Finally, the absorbance values of the cells were determined by using a microplate reader (Emax Precision, USA) at 570 nm. The

background absorbance of the well plate was measured and subtracted. The cytotoxicity was calculated by dividing the optical density (OD) values of treated groups (T) by the OD

values of the control (C) (T/C × 100%).

In vitro BMDMs study

To study the eATP-driven activation of NLRP3 inflammasome, bone marrow-derived macrophages (BMDMs) were primed with LPS (100 ng/mL) for 3 h. Then the BMDMs were treated with PBS, POM1, C-POM1, Met, C-Met, PMet and C-PMet at the metformin dosage of 200 μg mL^{-1} and the POM1 dosage of 100 μM, followed by stimulation with ATP (1 mmol/L). Proteins in total cell lysates and supernatants were detected by following mAbs: caspase1 p20 (Casper-1, AdipoGen Life Sciences), IL-1β (#12242, Cell Signaling Technology) and β-actin (ab8226, Abcam).

To explore the activation of AMPK pathway, BMDMs were cultured in a hypoxic cell incubator (2% O_2, 5% CO_2). Proteins in total cell lysates were harvested after indicated treatments and detected by following mAbs: AMPKα (#5831, Cell Signaling Technology), p-AMPKα (#50081, Cell Signaling Technology), HIF-1α (ab179483, Abcam), CD39 (ab227840, Abcam) and β-actin (ab8226, Abcam).

In vitro DC stimulation

For in vitro DC stimulation experiments, $1x10^5$ B16F10 cells were cultured with $1x10^6$ DC cells through a transwell co-culture system. After various treatments, DCs were stained with anti-CD45-APC/Cy7, anti-CD11c-FITC, anti-CD80-APC and anti-CD86-PE (Biolegend) antibodies, and then sorted by an FC500 flow cytometer

(Beckman-Coulter, USA). The supernatants from the co-culture system were also collected to measure the levels of TNF-α and IL-12p70.

The priming and effector function assay of T cells

For in vitro T cells proliferation assay, $CD8^+$ T lymphocytes from C57BL/6 mice spleen negatively selected by magnetic separation according to the protocol of $CD8a^+$ T Cell Isolation Kit (Miltenyi Biotec), were stained with the Cell Trace CFSE Cell Proliferation Kit (Invitrogen). CFSE-stained $CD8^+$ T cells were then cultured with above treated DCs for 72 h, and the proliferation of splenic T cells was measured by flow cytometry. As a parallel experiment, the effector function of incubated $CD8^+$ T cells was analyzed by measuring the expression of granzyme B (GZMB) and interferon-gamma (IFN-γ).

In vivo imaging

For in vivo fluorescence imaging, 2×10^5 B16F10 cells were injected into the right flank of the mice. When the tumor size reached around 100 mm^3, the mice were intravenously injected with the Cy5.5 labeled C-PMet (C-PMet@Cy5.5). The tumor accumulation of C-PMet@Cy5.5 was recorded on an IVIS Spectrum at different time intervals (3, 6, 12 and 24 h after the injection).

In vivo anti-tumor effects

For the B16F10 primary tumor model, B16F10 (2×10^5 cells each) suspended in 100 μL of PBS were injected into the right flank of the mice. After 7 days of subcutaneously implantation, mice were randomly divided into seven groups and administered with one of treatments: PBS, POM1, C-POM1, Met, C-Met, PMet and

C-PMet at the metformin dosage of 100 mg kg^{-1} and the POM1 dosage of 5 mg kg^{-1}, respectively. Afterwards, the growth of the tumors was carefully monitored. Tumor volume (V) was calculated by the formula: $V = 0.5 \times L \times W^2$, where L and W represent the length and width of the tumor. Mice were sacrificed on day 23 after tumor inoculation. The tumor tissues, peripheral blood and the major organ of mice were collected for analysis. The remaining mice were used for the survival study. Mice were sacrificed when the tumor burden exceeded 2 cm in diameter or if they lost up to 20% of their initial weight. Survival was evaluated from the first day of implantation until day 60.

In vivo toxicity evaluation

Blood samples were harvested from the B16F10 tumor-bearing mice to measure the levels of ALT (alanine aminotransferase), AST (aspartate aminotransferase), ALP (alkaline phosphatase), CRE (creatinine) and BUN (blood urea nitrogen). Major organs (hearts, livers, spleens, lungs and kidneys) were also collected and examined by H&E staining.

Transcriptomics study

For transcriptome analysis, 2×10^5 B16F10 cells were injected into the right flank of the mice. When the tumor volume reached about 100 mm^3, the mice were randomly divided into 2 groups and intravenously injected with PBS and C-PMet, respectively. After 3 days, tumor tissues were harvested and total RNA were extracted. The transcriptome analysis was performed in the Beijing Genomics Institute. All data were analyzed online with BGIseq500 platform (BGI-Shenzhen, China).

Tumor immune microenvironment analysis

For analysis of immune cells in primary tumors, Tumor tissues were digested with Tumor Dissociation Kit (Miltenyi Biotec) according to the manufacturer's protocol. After red blood cells lysis, single cell suspensions were stained with anti-CD45-APC/Cy7, anti-CD11b-PE, anti-F4/80-FITC, CD80-BV605 and CD206-APC fluorescence-labelled antibodies for M1 and M2-like macrophages. Stained with anti-CD45-APC/Cy7, anti-CD3-BV421 and anti-NK1.1-PE for NK cells. Stained with anti-CD45-APC/Cy7, anti-CD3-BV421, anti-CD4-PE and anti-CD8-APC for $CD4^+$ and $CD8^+$T cells. Stained with anti-CD45-APC/Cy7, anti-CD3-BV421, anti-CD8-APC and anti-Ki67-PE for Ki67-positive $CD8^+$ T cells. Stained with anti-CD45-APC/Cy7, anti-CD3-BV421, anti-CD8-APC and anti-granzyme B-PE for GZMB-positive $CD8^+$ T cells. Stained with anti-CD45-APC/Cy7, anti-CD3-BV421, anti-CD8-APC and anti- IFN-γ-PE for IFN-γ-positive $CD8^+$ T cells. Stained with anti-CD45-APC/Cy7, anti-CD3-BV421, anti-CD4-PE and anti-Foxp3-APC for regulatory T cells (Tregs). Stained with anti-CD45-APC/Cy7, anti-CD11b-PE and anti-Gr1-APC for myeloid-derived suppressor cells (MDSCs). Stained with anti-CD45-APC/Cy7, anti-CD3-BV421, anti-CD8-APC and anti-CD39-PE for CD39-positive $CD8^+$ T cells. Stained with anti-CD45-APC/Cy7, anti-CD3-BV421, anti-NK1.1-PE and anti-CD39-APC for CD39-positive NK cells. For DCs activation analysis in tumor-draining lymph nodes, single cell suspensions were stained with anti-CD45-APC/Cy7, anti-CD11c-FITC, anti-CD80-APC and anti-CD86-PE. Furthermore, infiltrating T lymphocytes and

expression of HIF-1α in primary tumors were analyzed by immunofluorescence staining.

In vivo anti-metastasis study

For the lung metastasis model, 2×10^5 B16F10-Luc cells in 100 μL PBS were subcutaneously injected into the right flank of C57BL/6 mice (day -8). 7 days (day -1) later, the mice were intravenously injected with 1×10^5 B16F10-Luc cells and randomly divided into four groups: PBS (G1), C-PMet (G2), PD1 (G3) and C-PMet+PD1 (G4). On 1 day (day 0) after B16F10-Luc intravenous injection, the mice in G2 and G4 were iv injected with C-PMet. For G3 and G4, the mice were intravenously injected with 0.2 mg PD-1 (clone RMP1-14, BioXCell) monoclonal antibody in 150 μL PBS on day1, 4 and 7. The mice were sacrificed on day 21 and all of the lungs were harvested, photographed, fixed, and sections were taken for H&E staining. The metastasis nudes of all mouse were counted to evaluate the anti-metastasis effect. Lung metastasis was also detected by ex vivo luciferase based noninvasive bioluminescence imaging system. For analysis of immune cells in lung metastatic tumor, lung tissues were digested with Tumor Dissociation Kit (Miltenyi Biotec) according to the manufacturer's protocol. After red blood cells lysis, single cell suspensions were stained with anti-CD45-APC/Cy7, anti-CD3-BV421, anti-CD4-PE and anti-CD8-APC for $CD4^+$ and $CD8^+$T cells. Stained with anti-CD45-APC/Cy7, anti-CD3-BV421 and anti-NK1.1-PE for NK cells.

For tumor distant metastasis model, 2×10^5 B16F10-Luc cells in 100 μL PBS were subcutaneously injected into the right flank of C57BL/6 mice (day -8). 7 days

(day -1) later, the mice were injected with 2×10^5 B16F10-Luc tumor cells into the left flank as the distant tumors and randomly divided into four groups: PBS (G1), C-PMet (G2), PD1 (G3) and C-PMet+PD1 (G4). On 1 day (day 0) after B16F10-Luc injection, the mice in G2 and G4 were iv injected with C-PMet. For G3 and G4, the mice were intravenously injected with 0.2 mg PD-1 (clone RMP1-14, BioXCell) monoclonal antibody in 150 μL PBS on day1, 4 and 7. Afterwards, the growth of the primary and distant tumors were carefully recorded. Tumor volume (V) was calculated by the formula: $V = 0.5 \times L \times W^2$, where L and W represent the length and width of the tumor. The distant tumor was also observed using an in vivo bioluminescence imaging system. For analysis of immune cells in distant tumor, tumor tissues were digested with Tumor Dissociation Kit (Miltenyi Biotec) according to the manufacturer's protocol. After red blood cells lysis, single cell suspensions were stained with anti-CD45-APC/Cy7, anti-CD3-BV421, anti-CD4-PE and anti-CD8-APC for $CD4^+$ and $CD8^+$T cells. Stained with anti-CD45-APC/Cy7, anti-CD3-BV421 and anti-NK1.1-PE for NK cells.

In vivo anti-rechallenge study

To evaluate the long-term immune protection after the treatment with our strategy, tumor rechallenge model was established. Briefly, 2×10^5 B16F10 cells in 100 μL PBS were subcutaneously injected into the right flank of C57BL/6 mice (day -7). 7 days (day 0) later, the mice were randomly divided into four groups: Surgery (G1), C-PMet (G2), PD1 (G3) and C-PMet+PD1 (G4). For G2 and G4, the mice in G2 and G4 were iv injected with C-PMet three times for a week until the tumors were eliminated. For

G1 and G3, the primary tumors were removed by surgical resection. On day 40, 2×10^5 B16F10 cells in 100 μL PBS were subcutaneously injected into the right flank of C57BL/6 mice to establish the tumor rechallenge model. For G3 and G4, the mice were intravenously injected with 0.2 mg PD-1 (clone RMP1-14, BioXCell) monoclonal antibody in 150 μL PBS on day 41, 44 and 47. Afterwards, the growth of the rechallenged tumor was carefully recorded. Tumor volume (V) was calculated by the formula: $V = 0.5 \times L \times W^2$, where L and W represent the length and width of the tumor. For memory T cells analysis, spleens were excised and stained with anti-CD45-APC/Cy7, anti-CD3-BV421, anti-CD8a-APC, anti-CD44-PE and anti-CD62L-BV605 fluorescence-labelled antibodies according to protocols.

Patient-derived organotypic slice culture

Fresh colon tumor tissues and blood were obtained from one colon cancer patient undergoing primary surgical treatment at the Zhongnan Hospital of Wuhan University. The study was approved by the Protection of Human Subjects Committee of Zhongnan Hospital. Tumor tissues were cut into thin slices at the size about 6×6×2 mm and randomly divided into twelve parts. Then the tumor slices were incubated with PBMCs (1×10^6 cells mL^{-1}) obtained from blood by Ficoll density gradient separation in 8 mL RPMI 1640 medium containing 10% FBS in the presence of PBS, C-PMet derived from HCT116 colon cancer cells, anti-human PD-1 antibody, or C-PMet plus anti-human PD-1 antibody, respectively. After incubation at 37 °C for 36 h, the tumor slices were washed by PBS for three times, cut into small pieces and digested with 1.5 mg/mL collagenase IV and 0.2 mg/mL DNase I in RPMI 1640

medium at 37 °C for 1 h. The samples were then passed twice through a 70 μm cell strainer to obtain single-cell suspensions. Tumor cells were gated by anti-human CD45-FITC (Biolegend, cat. No 304006, clone HI30) negative and stained with anti-Annexin V (Biolegend, cat. No 640920) and PI to analyze the apoptosis. For tumor associated macrophages (TAMs) analysis, single cell suspensions were stained with anti-human CD45-FITC (Biolegend, cat. No 304006, clone HI30), anti-human CD11b-APC (Biolegend, cat. No 301350, clone ICRF44), anti-human CD14-PE (Biolegend, cat. No 301806, clone M5E2), anti-human CD80-PE/Cy7 (Biolegend, cat. No 305218, clone 2D10) and anti-human CD206-BV421 (Biolegend, cat. No 321126, clone 15-2). For NK cells analysis, single cell suspensions were stained with anti-human CD45-FITC (Biolegend, cat. No 304006, clone HI30), anti-human CD3-PE/Cy7 (Biolegend, cat. No 344816, clone SK7) and anti-human CD56-APC (Biolegend, cat. No 398806, clone QA18A21). For CD8+ T cell infiltration and effector function analysis, single cell suspensions were stained with anti-human CD45-FITC (Biolegend, cat. No 304006, clone HI30), anti-human CD3- PE/Cy7 (Biolegend, cat. No 344816, clone SK7), anti-human CD8a-PE (Biolegend, cat. No 300908, clone HIT8a). All antibodies were diluted according to protocols. The cells were then washed by PBS that containing 1% FBS and finally analyzed by FACS.

Statistical analysis

All results in this study were presented as the mean ± s.d. One-way analysis of variance (ANOVA) was conducted for multiple comparisons. The Kaplan-Meier method was used to obtain survival curves and the log-rank test was conducted to

compare survival benefit. All data analyses were carried out using GraphPad Prism 8.0 software.

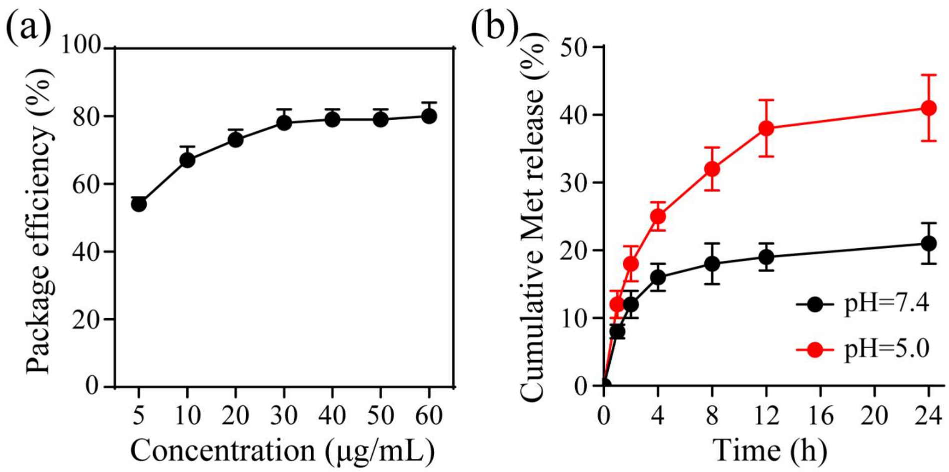

Figure S1. (a) Electroporation efficiency under different Met concentration. Data are presented as mean ± s.d. (n = 3). (b) Drug release of C-Met in PBS at different pH values. Data are presented as means ± s.d. (n = 3).

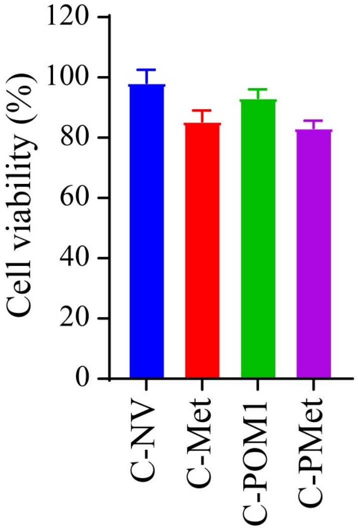

Figure S2. The viability of the B16F10 cells treated with C-NV, C-Met, C-POM1 and C-PMet.

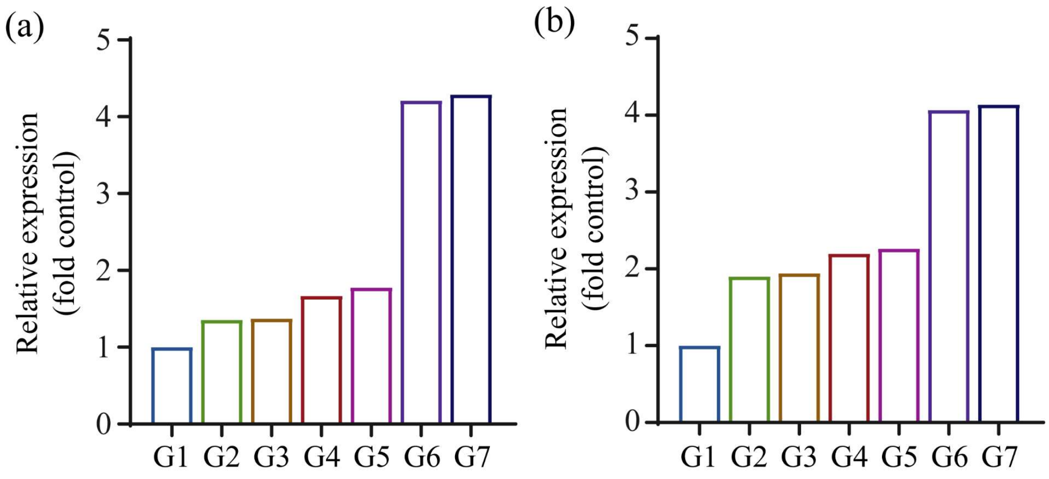

Figure S3. The relative protein expression levels of GZMB (a) and IFN-γ (b) from T cells after different treatment were detected by western blot analysis.

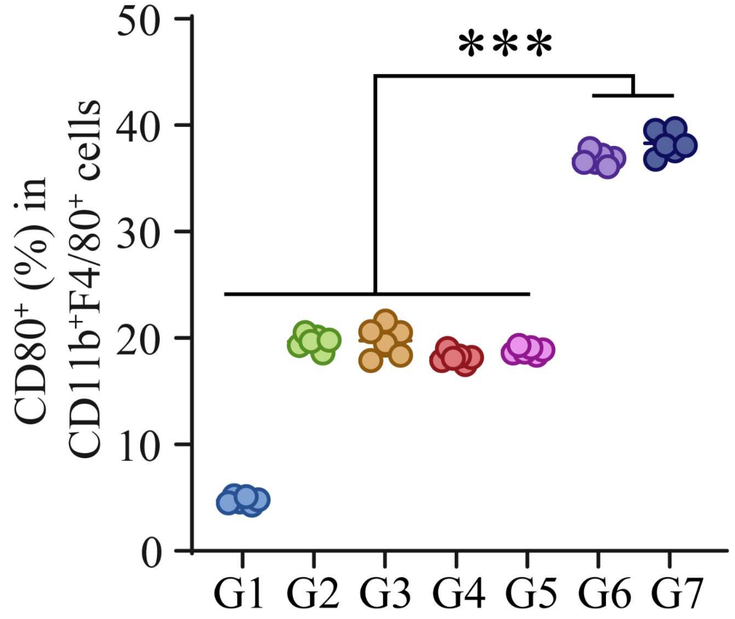

Figure S4. Quantification of M1-like ($CD45^{+}CD11b^{+}F4/80^{+}CD80^{+}$) macrophages in BMDMs after different treatments. Data are presented as mean ± s.d. (n = 6). *** p< 0.001.

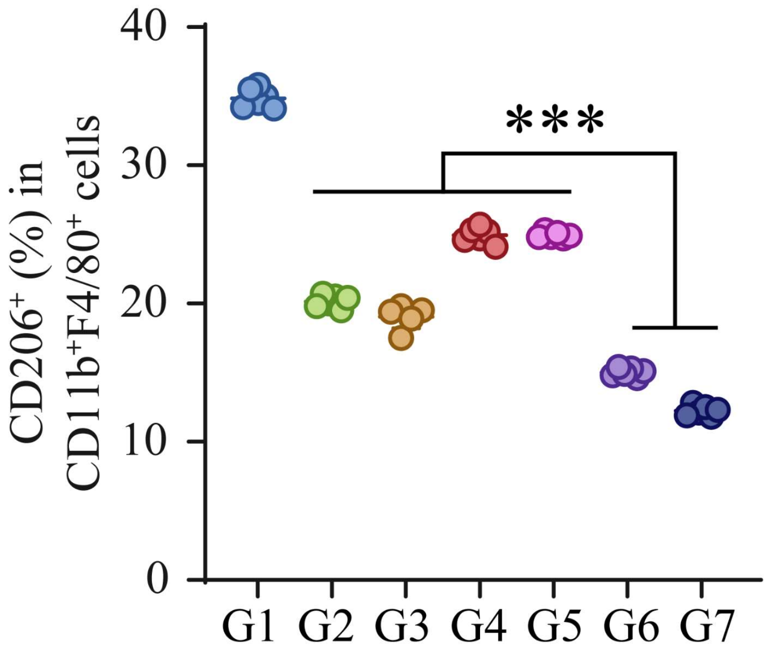

Figure S5. Quantification of M2-like ($CD45^{+}CD11b^{+}F4/80^{+}CD206^{+}$) macrophages in BMDMs after different treatments. Data are presented as mean ± s.d. (n = 6). *** p< 0.001.

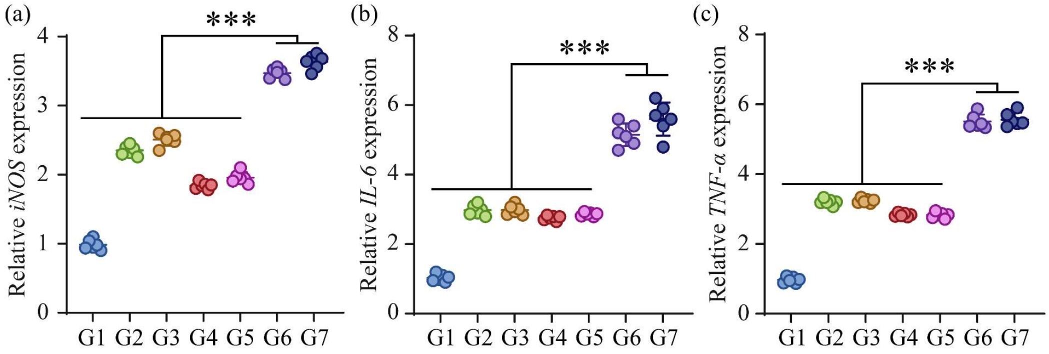

Figure S6. mRNA expression levels of *iNOS* (a), *IL-6* (b), and *TNF-α* (c) in BMDMs after different treatments. Data are presented as mean ± s.d. (n = 6). *** $p < 0.001$.

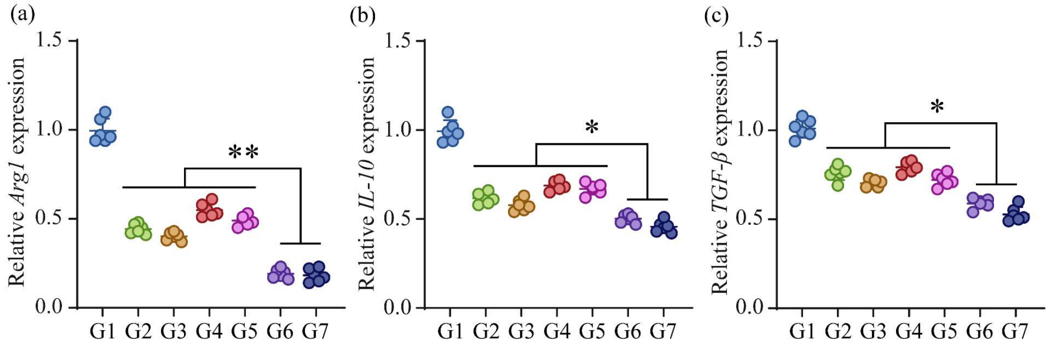

Figure S7. mRNA expression levels of *Arg1* (a), *IL-10* (b), and *TGF-β* (c) in BMDMs after different treatments. Data are presented as mean ± s.d. (n = 6). * $p < 0.05$, ** $p < 0.001$.

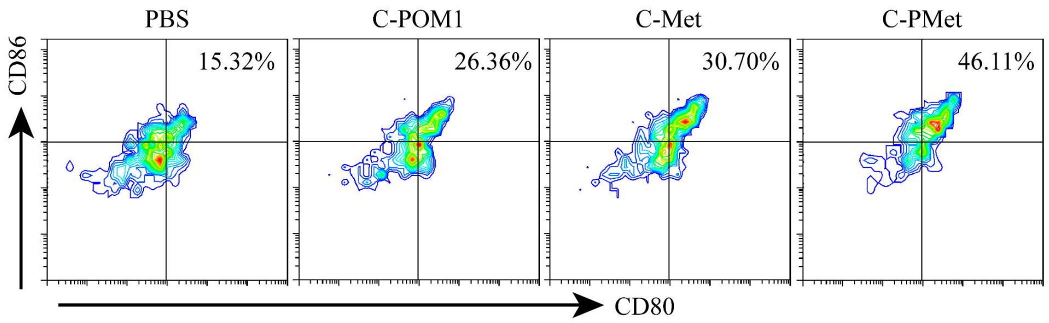

Figure S8. Representative flow cytometry plots showing BMDCs maturation rate (gated on $CD11c^{+}CD80^{+}CD86^{+}$).

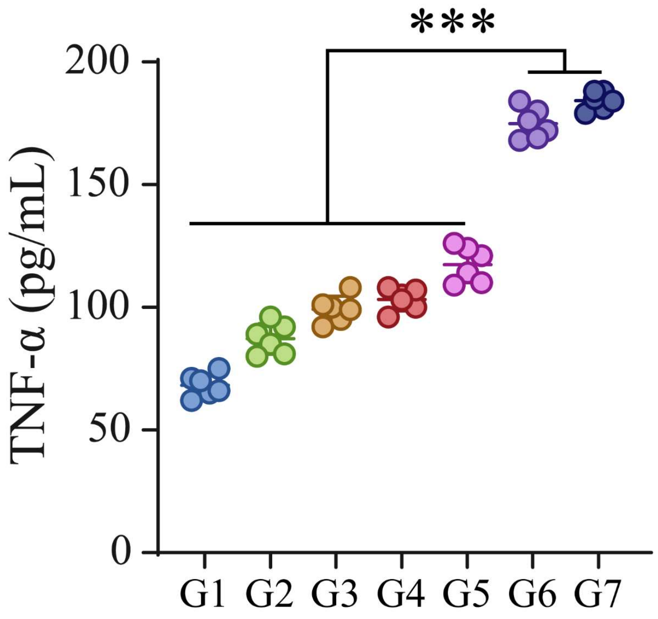

Figure S9. Secretion of TNF-α from the supernatant of BMDCs. Data are presented as mean ± s.d. (n = 6). *** $p < 0.001$.

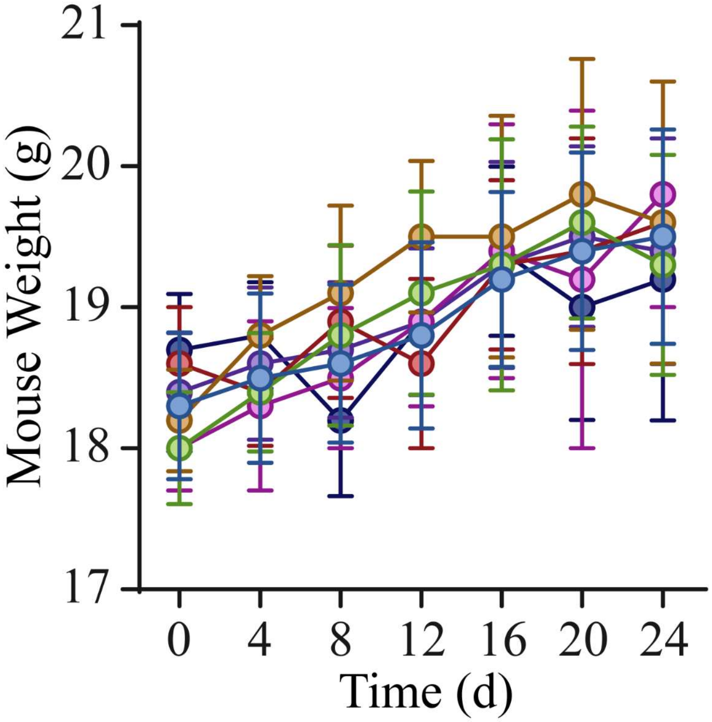

Figure S10. Mouse body weight change curves after various treatments.

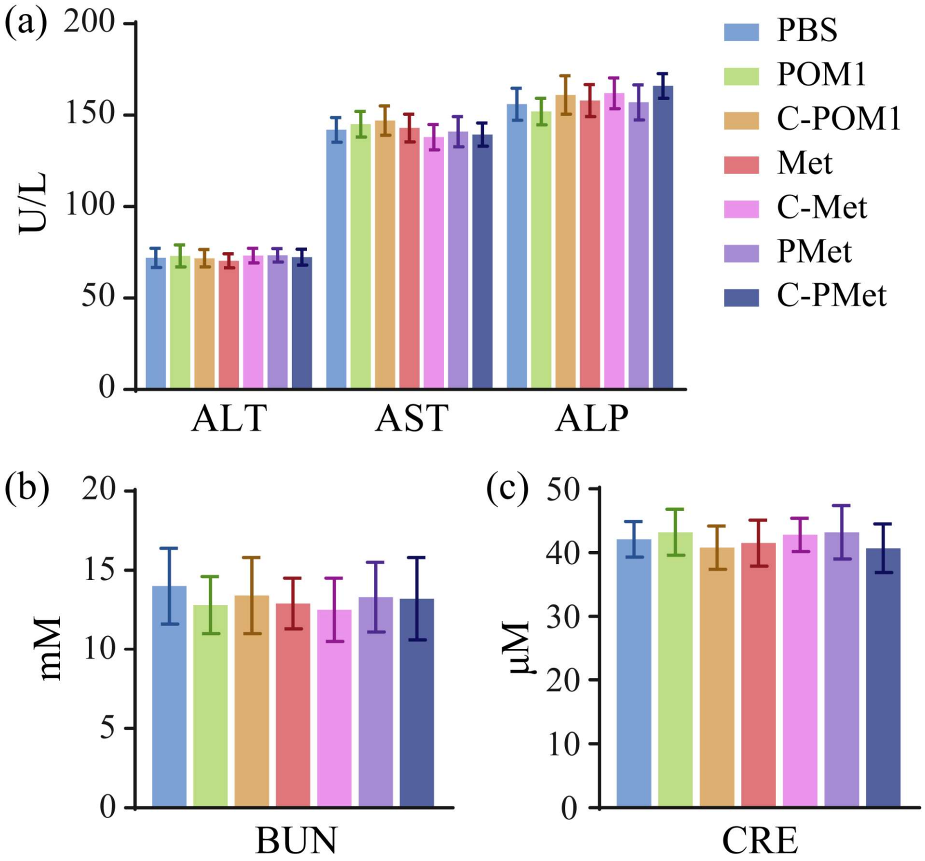

Figure S11. The measured images of three important hepatic indicators (i.e., ALT, AST, and ALP) and two indicators for kidney functions (i.e., BUN and CRE).

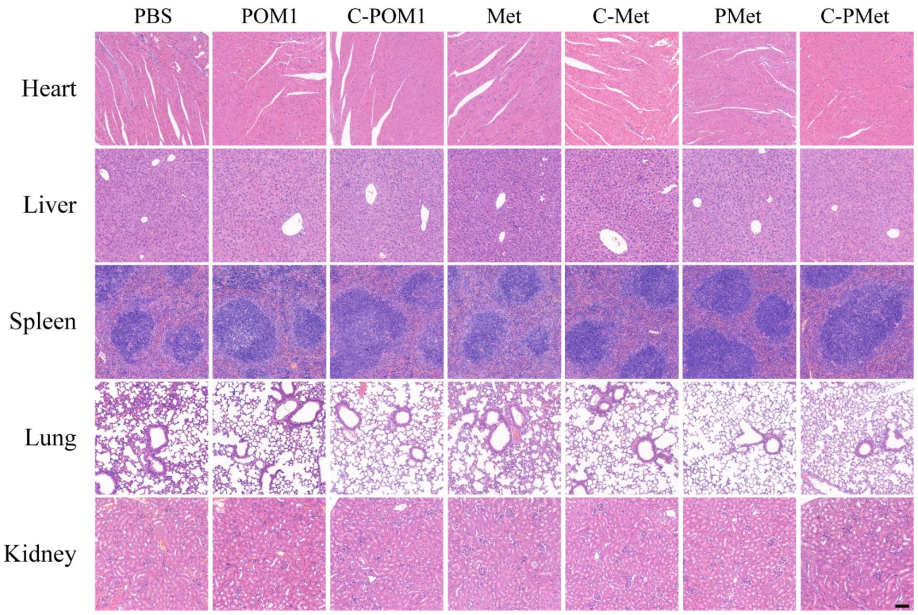

Figure S12. The images of H&E staining major organs from mice after various treatments. Scale bar = 100 μm.

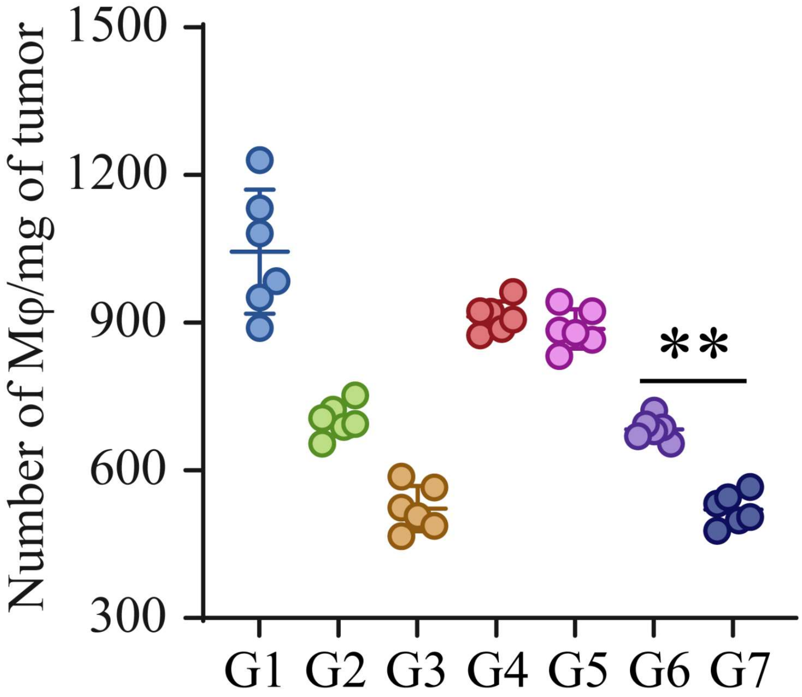

Figure S13. Numbers of B16F10 tumor-infiltrating macrophages after different treatments. Data are presented as mean ± s.d. (n = 6). ** p< 0.01.

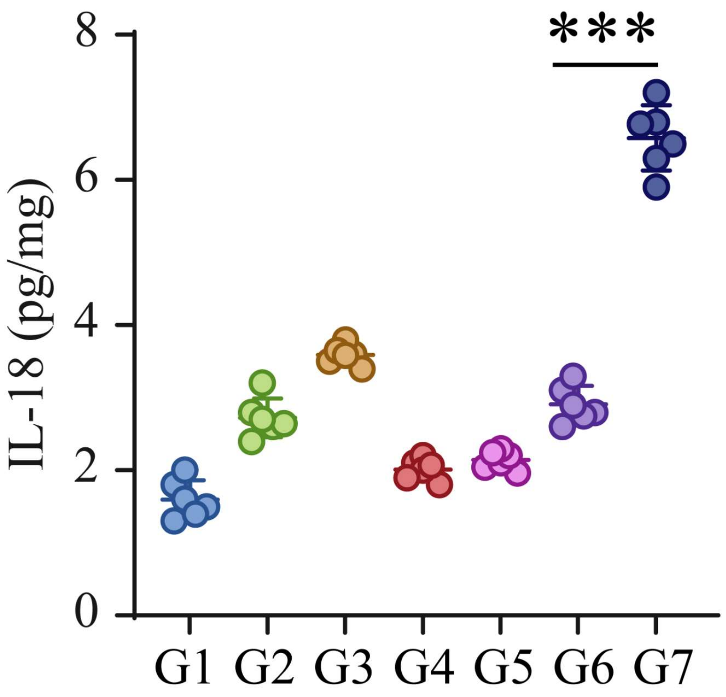

Figure S14. Inflammasome IL-18 production in B16F10 tumors after different treatments. Data are presented as mean ± s.d. (n = 6). *** p< 0.001.

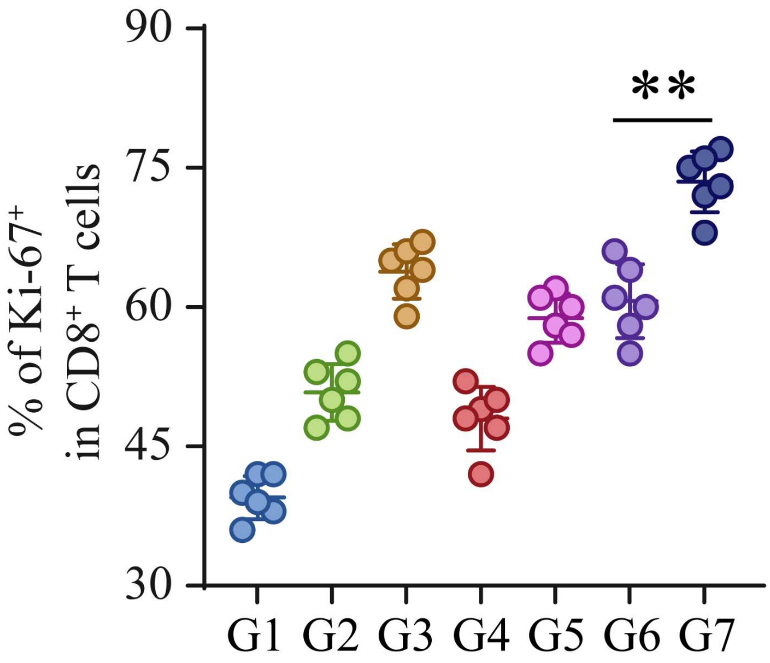

Figure S15. Percentages of $CD8^+$ T cells expressing Ki-67 with different treatment indicated. Data are presented as mean ± s.d. (n = 6). ** $p < 0.01$.

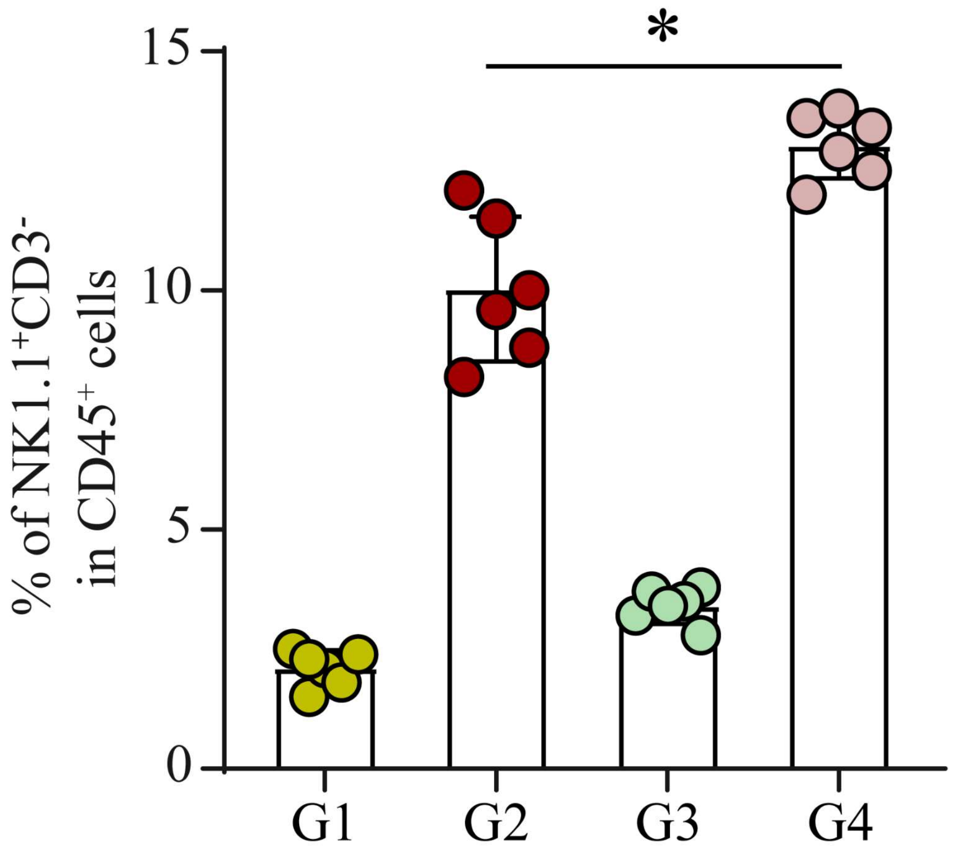

Figure S16. Quantitative analysis of NK cells (NK1.1$^+$CD3$^-$) in distant tumor tissues (gated on CD45$^+$ cells). Data are presented as mean ± s.d. (n = 6). * $p < 0.05$.

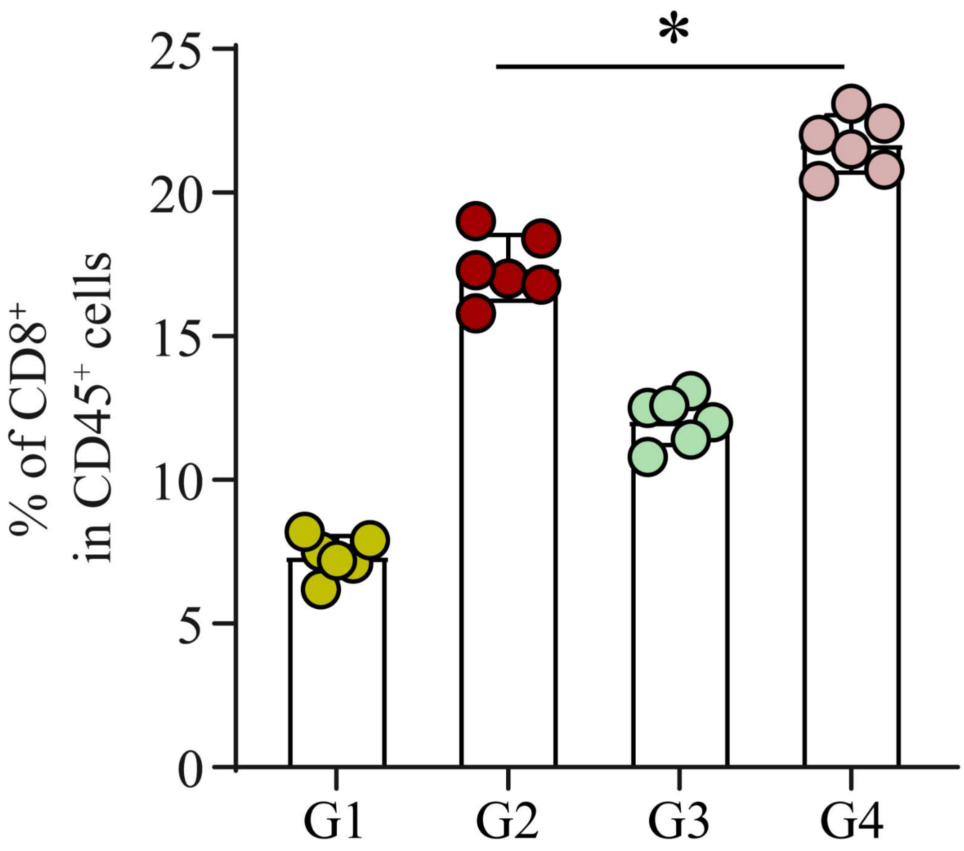

Figure S17. Quantitative analysis of $CD8^+$ T cells in distant tumor tissues (gated on $CD45^+$ cells).

Data are presented as mean ± s.d. (n = 6). * $p< 0.05$.

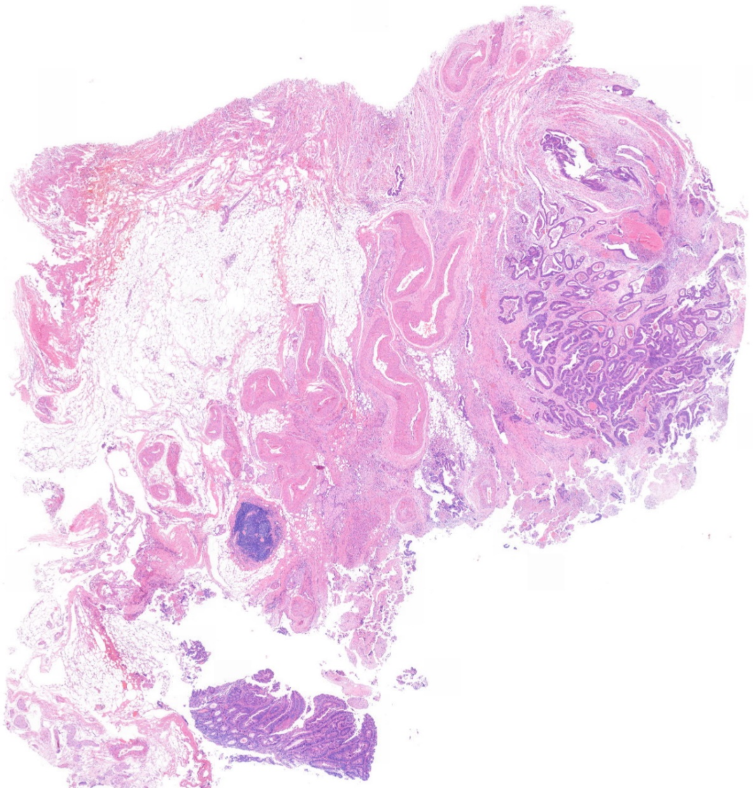

Figure S18. H&E staining of the patient-derived colon tumor.

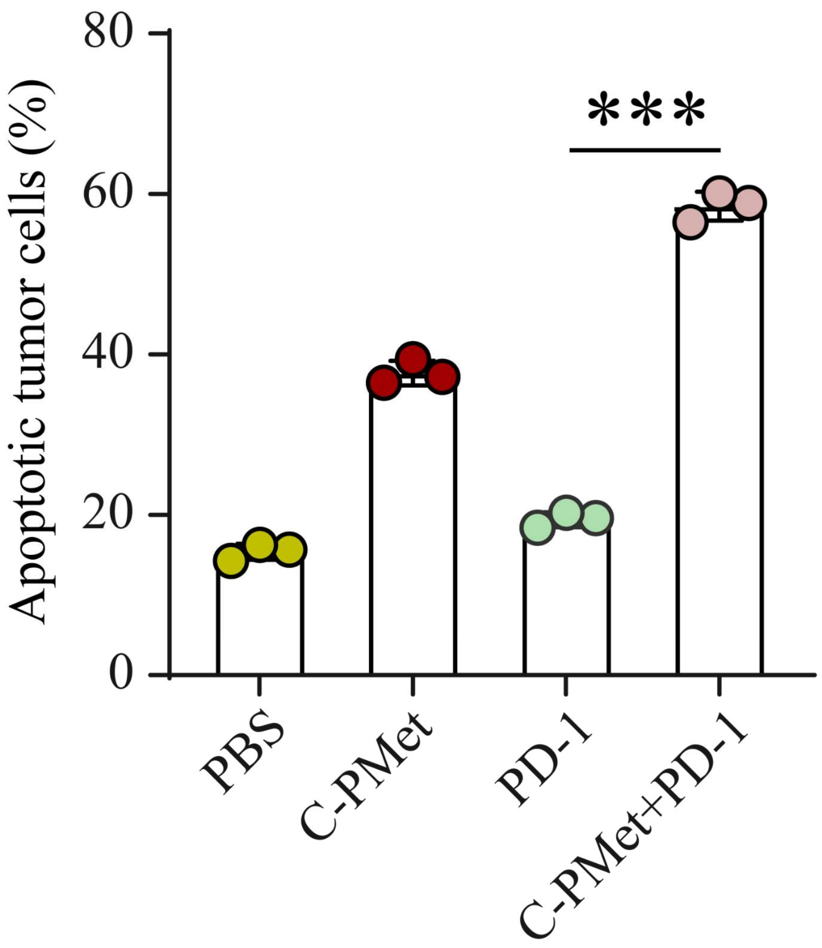

Figure S19. The ratio of apoptotic tumor cells after the tumor slices from patient-derived tumor after different treatments. Data are presented as mean ± s.d. ($n = 3$). *** $p< 0.001$.
